# Oncoimmunology Meets Organs-on-Chip

**DOI:** 10.3389/fmolb.2021.627454

**Published:** 2021-03-26

**Authors:** Fabrizio Mattei, Sara Andreone, Arianna Mencattini, Adele De Ninno, Luca Businaro, Eugenio Martinelli, Giovanna Schiavoni

**Affiliations:** ^1^Department of Oncology and Molecular Medicine, Istituto Superiore di Sanità, Rome, Italy; ^2^Department of Electronic Engineering, University of Rome Tor Vergata, Rome, Italy; ^3^Interdisciplinary Center for Advanced Studies on Lab-on-Chip and Organ-on-Chip Applications (ICLOC), University of Rome Tor Vergata, Rome, Italy; ^4^Institute for Photonics and Nanotechnologies, Italian National Research Council, Rome, Italy

**Keywords:** cancer immunology, microfluidic device, Cell on Chip, Organ on Chip, tumor microenvironment, personalized medicine, Oncoimmuno chip, human on chip

## Abstract

Oncoimmunology represents a biomedical research discipline coined to study the roles of immune system in cancer progression with the aim of discovering novel strategies to arm it against the malignancy. Infiltration of immune cells within the tumor microenvironment is an early event that results in the establishment of a dynamic cross-talk. Here, immune cells sense antigenic cues to mount a specific anti-tumor response while cancer cells emanate inhibitory signals to dampen it. Animals models have led to giant steps in this research context, and several tools to investigate the effect of immune infiltration in the tumor microenvironment are currently available. However, the use of animals represents a challenge due to ethical issues and long duration of experiments. Organs-on-chip are innovative tools not only to study how cells derived from different organs interact with each other, but also to investigate on the crosstalk between immune cells and different types of cancer cells. In this review, we describe the state-of-the-art of microfluidics and the impact of OOC in the field of oncoimmunology underlining the importance of this system in the advancements on the complexity of tumor microenvironment.

## Introduction

Microfluidic platforms represent an emergent and promising technology for life science research aiming to reproduce specific biological environments that recapitulate the *in vivo* scenario with higher fidelity with respect to conventional *in vitro* techniques (Jiménez-Díaz et al., 2019; Wang L. et al., 2019). They are generically composed of a device coupled to pump circuits or wells for liquid circuits and a microscope to make real time acquisitions during the experiment. These systems represent useful tools for multiple purposes, spacing in all the areas of life sciences, and imply the involvement of multidisciplinary sciences. Indeed, while biologists and physicians pose the scientific problem, physicists conceive the devices and, finally, statisticians or mathematicians extrapolate results by processing the large array of data obtained by microfluidic-based studies.

An emerging field of application of microfluidic chips comprise the study of complex cell microenvironments, such as the tumor microenvironment (TME) and how immune cells and tumor cells interact within it. The TME is a complex environment composed by multiple cell types, including blood vessels, immune cells, stromal cells, fibroblasts, and by soluble factors, which closely and constantly interact with tumor cells, thereby determining the fate of tumor progression (Schiavoni et al., 2013; Anderson and Simon, 2020). The advancements of molecular and cellular profiling technologies has gained key information on how distinct immune cell populations interact with the tumor and the other TME components (Liu et al., 2019; Davidov et al., 2020). However, these methodologies do not allow to investigate complex interactions, processes and mechanisms behind the full TME functioning. The detailed knowledge of the tissue microenvironment and the associated dynamics led to the development of Tissue-On-Chip (TOC) platforms. These systems aim at recapitulating the specific functions of tissues on microfluidic devices, i.e., blood, bone marrow, lung, liver, just to cite some examples. However, they are often defined as Organs-On-Chip (OOC) to indicate the organ they aim to recapitulate (Li et al., 2019b). The OOC definition is optimal for the majority of advanced multicellular living systems and can be extended to particularly heterogeneous organs such as tumors. The use of TOC definition is particularly recommended to mimic cell-cell interactions on-chip in several tissues with undefined compartmentalization or boundaries, such as blood, bone-marrow and specific hematopoietic niches (Aleman et al., 2019). TOCs can also be employed for the generation of microfluidic systems supplemented by spheroids (Duarte Campos et al., 2020). Spheroids are indeed entities similar to organs but not completely resembling them. This concept can also be extended to tumor spheroids when the cells forming the spheroid are of malignant nature.

Over time, the definition OOC has been mostly preferred to TOC since OOC comprise all tissue types. The advent of OOC has boosted the research on cell-cell interactions in basilar and advanced cell systems. This platform proved to be fundamental for the study of cell-cell interaction and migratory behaviors upon specific perturbations (Kim and Haynes, 2013; Menon et al., 2014). Moreover, OOC can be considered as a “simplified organ” potentially useful to bypass diverse problems intrinsic for *in vitro* and *in vivo* experiments. In addition, OOCs better recapitulate multicellular systems (e.g., an organ) and are ideal tools to study dynamically complex organs, including the TME. In other words, the use of OOCs aim to achieve an appropriate degree of simplification by reconstituting an *in vivo* system to an *in vivo*-like model of study. In these settings, it is possible to follow the system’s components without altering their original function. Thus, OOC systems can be classified as advanced *in vitro* smart platforms evolved from *in vitro* and *in vivo* scientific knowledge (Boussommier-Calleja et al., 2016).

In this review, we describe the state-of-the-art of microfluidic devices and explain how and why the advent of microfluidic platforms markedly impacted the study of multicellular systems with emphasis on their employment for oncoimmunology studies.

## From Pioneers of Microfluidics to Advanced OOC Systems

The term microfluidics defines all the systems, simple or advanced, where a fabrication process that generates micrometers-sized scale channels is the key component of that system. More than 50 years ago, researchers perceived that microfluidic systems can dramatically impact the study of chemical compounds and cells. Of note, the advances in materials technology has certainly facilitated the expansion of microfluidic systems. These pioneer research studies evidence how the distinction between the terms Lab-on-a-Chip (LC) and Cell-on-a-Chip (CC) is generally represented by the nature of the elements investigated within the device. In general, LC covers all studies in which microdevices are used alongside with chemicals or proteins or other substances such as lipids. CC systems are employed for live cell investigations and OOC platforms are usually employed to follow simple or complex multicellular environments. All the three terms, LC CC and OOC, can sometimes overlap, due to lack of standardization of these three definitions. This adds further grade of complexity to the problem of searching univocal definitions for the microfluidic chip-based systems. A history of the birth of the first microfluidic systems and how they became advanced platforms such as OOC is delineated, with emphasis on the use of OOC in an oncoimmunology context.

### First Approaches to Microfluidics Systems: LC

The first literature data on microfluidic approaches is a paper by Lew and Fung (1969). They were the first to outline the concept of simulation theory in the attempt to mimic the fluidic dynamics of the blood flow circulation and bronchioles air flow simulations within lung tissue. They asserted that the flow generated by the uniform axial velocity is relevant for blood flow in branching blood vessels and for the motion of plasma in the capillary blood vessels. This theoretic microtube system, with a diameter equal or less to 100 μm, is generated by complex algorithms governing the fluid dynamics. These Authors were the first to introduce the notion of inlet/outlet in the liquid flow simulations for live systems such as blood circulation and lung bronchioles. This fluidic model has been repurposed by an independent laboratory 20 years later (Xue and Fung, 1989). The importance of this first simulation study lies on the notion that the main role of microfluidic devices is to recapitulate the multicellular living systems in their multifaceted aspects, including biochemical cues behind the cell-cell communication. This key milestone could not be reached without the LC systems. LCs proved decisive for a better knowledge of cell-cell signaling, mimicking specific inner events including those relative to immune cells.

The first use of a LC for investigative purposes on living (eukaryotic) cells was made by Esch et al. (2001a, b) for the detection of *Cryptosporidium Parvum*, a waterborne pathogen. They used a method relying on the ability of these bacteria to release some typical and exclusive DNA sequences that are PCR-amplified within *ad hoc* fabricated microfluidic chips. These studies demonstrated that microdevices can be used to investigate the effects of chemicals and proteins released from bacteria and cells. For these reasons, the majority of scientists coined the term LC to evidence how these devices can be conceived as actual miniaturized laboratories. Such mini-labs are focused at investigating specific substances such as chemicals or proteins, but are not suitable for living cells.

In 2012, Farmer and co-Workers generated a LC microfluidic system to recapitulate a red blood cell. They were the first to mimic a cell model, even though not a living cell, with microfluidic approaches (Farmer et al., 1988). This first study in a microfluidic cartridge has certain been facilitated by the non-living nature of the system. This work can be considered as one of the first attempts to obtain a primitive cell-mimicking system. The first microfluidic system to immobilize particular types of proteins came on the 1990s, with a study from Johnsson et al. (1991). This research describes how microfluidic systems can be applied in the field of sensors engineering. On the other hand, it represents one of the first studies reporting a complete microfluidic chip fabrication process as an important part of device design.

A relevant giant step on the creation of LCs as sensors useful for cancer prevention comes from an elegant paper of Bahavarnia and co-Workers. Here a paper-based immunosensor has been generated, coupled to a fluidic chamber to propagate the fluid toward the sensor area in which the CA125 protein will be detected by an ELISA-based assay (Bahavarnia et al., 2019). Similar approaches based on the immunosensors have been described in literature, useful to study apoptotic activity (Dang et al., 2019) or soluble immune checkpoint inhibitors (Reza et al., 2019) in biological fluids or clinical samples. These studies represent interesting examples of LCs to be applied for oncoimmunology applications.

Lab-on-Chip are generally appropriate to study specific mechanical activities or fluidic phenomena, often to be addressed to specific living cell mimicry events. With the development of more advanced microfluidic fabrication techniques and with the advent of sophisticated biotechnology tools, LC platforms have evolved into complex systems to investigate on some features of single cells. For instance, an LC platform was developed with the aim to identify the disparate antigen specific antibodies secreted by single B-lymphocytes during the development of innate immune responses (Gérard et al., 2020). Fabrication technologies have also allowed implementation of LC into efficient and affordable biosensors. LC biosensors have been recently developed and optimized to be applied in oncoimmunology. A clear example is represented by LC biosensors to detect cancer biomarkers, where the main LC biomaterial is represented by paper and nano-inks (Bahavarnia et al., 2019). Despite the discovery of disparate materials well suited for biological studies, the use of Polydimethylsiloxane (PDMS)-based techniques still remains the elected methodology to fabricate all microfluidic devices, including LC biosensors. Next generation chips will be represented by microfluidic platforms where the use of the PDMS may be supplemented, rather than superseded, by the use of other biomaterials needed for chip optimization.

### Beyond the LC Platforms: The CC Systems

While *in vitro* Two dimensional (2D) and Three dimensional (3D) culture systems are useful to study specific cell features, the advent of LC equipped investigators with versatile tools to monitor cell behaviors in real time. This began the initiation of the CC era.

In 1997 Li and co-Workers carried out a glass microfluidic CC system suitable to monitor the mobilization of red blood cells, yeast cells as well as *Escherichia Coli* bacteria in a device composed by several interconnected channels. This chip constituted one of the first approaches to study living cells by means of a device composed by multiple substructural units (Li and Harrison, 1997). A first study analyzing cell docking and alignment was produced by Yang and co-Workers on 2002 with human hematological HL-60 tumor cell lines (Yang et al., 2002). Another report where cells were monitored in a CC system employed a feochromocytoma derived PC12 cell line (Huang et al., 2004) in the attempt to follow their mobilization features. The development of CC research with tumor cells has been facilitated by the fact that these cells do not require stringent factors for their growth, compared to primary cells (Zielinski et al., 2017). For this reason, tumor cells can be easily studied on chip, in particular for long-term time points. In 2005, primary cells were successfully studied on a CC system in an interesting work focused at recapitulating the cardiac myocyte contraction (Li and Li, 2005). This and other studies (Ho et al., 2006; Kokkinos et al., 2008; Zhao et al., 2012) constitute some representative examples of the first primitive studies carried out in CC systems. All of them utilized a single cell type with simple analyses and evaluations, such as mobilization within microscale-sized spatial units and brief time intervals.

The first attempt to study a specific immune cell population on chip was reported in 2006 by Matsumura and co-Workers, who investigated the ability of macrophages to start and sustain zymosan-induced phagocytosis in fibronectin-coated CC by time-lapse microscopy. This represented a first study in which a functional immune event has been reproduced on chip (Matsumura et al., 2006). Another interesting CC system has been generated by Kang and co-Workers. Their structure is represented by a chamber where bead-labeled blood cells flow upon a micromagnetic field. This field has a key role in capturing rare circulating tumor cells or metastatic cells (Kang et al., 2012). The particularity of this microfluidic chip is the possibility to culture a single cell type by flushing away the other undesired cells, such as circulating leukocytes.

An important phenomenon to be investigated in the field of oncoimmunology is the migratory behavior of immune cells toward the primary tumor site. Thus, it is not surprising that researchers pay particular attention on the study of immune cell migration on chip. A first example of such studies was provided by Molino and co-Workers. They described a CC system properly designed to follow leukemic cell migration toward an oil droplet (Molino et al., 2016). For some aspects, these droplets resemble immune cell moving in the CC microchannel. This work is a clear example of how CC studies inspired experimental implementations to design the more complex OOC systems, in order to study the interaction between immune cells and cancer within *ad hoc* fabricated microfluidic devices.

### Putting Different Cell Types on a Chip: The OOC Platforms

The term OOC underlines the attempt to load different cell types residing organs or multicellular complexes inside a microfluidic device, often coupled to sophisticated microscopy systems. In general, such a definition fits also for chips loaded with at minimum two different cell populations, even if these are not able to develop in complex multicellular systems (Sackmann et al., 2014; Reardon, 2015; Chen P. et al., 2020). The simpler the OOC is the easier cells are monitored within it.

A first approach to study immune system on chip has been developed by Whitesides and co-Workers. They created a simple OOC platform to co-culture two different types of macrophage-like cells (Wong et al., 2008). In this report, BAC1.2F5 and LADMAC macrophage cell lines were loaded in separated chambers of the microdevice. Then, the production of the colony stimulating factor-1 (CSF-1), released by LADMAC and required by BAC.2F5 cells for survival, was monitored over time. This simple OOC prototype can also be used to study the mutual crosstalk between immune and cancer cells.

The first attempt to design a microfluidic platform employing different cell types on chip was reported by Shuler and co-Workers on 2009. They adapted a microfluidic chip for simultaneous co-culture of tumor cells (colon cancer cells HCT-116 or Hepatoma cells HepG2, embedded in hydrogels) with liver and bone marrow cells (Sung and Shuler, 2009). This system allowed to elegantly test the cytotoxicity of a specific anticancer agent on tissues when directly delivered on chip. Despite allowing the simultaneous loading of different cells, this OOC system does not allow to study the crosstalk between loaded cells, such as migration of bone-marrow cells toward the tumor in response to the drugs. In a study investigating the crosstalk between immune cells and cancer cells, Businaro and co-Workers successfully attempted to investigate on the mutual behavior of melanoma and immune cells (Businaro et al., 2013). This approach was inspired by the difficulty to study the events behind the immune cell infiltration inside the TME, and represents a new dawn for the oncoimmunology on chip. Previous work *in vivo* showed that accelerated B16.F10 melanoma progression in non-immunocompetent mice was the result of altered cytokine/chemokine production and impaired infiltration of different murine immune cell subsets in the TME (Mattei et al., 2012). Based on these observations, microfluidic devices were employed as a simple OOC system to evaluate gradient-dependent interactions between B16.F10 melanoma cells and spleen-derived immune cells from naïve immunocompetent vs non-immunocompetent mice. In detail, a PDMS microdevice constituted by a 1,000 μm-sized central chamber connected to two side chambers by two arrays of 12 μm-sized microchannels has been used. The two side chambers were loaded with spleen cells (from immunocompetent or IRF-8 deficient mice) and B16.F10 melanoma cells. Time-lapse video recording was carried out over a 48 h-period in a region comprising the B16 side chambers and the adjacent microchannels. The results evidenced a mutual crosstalk between B16 cells and spleen cells. Specifically, in OOC loaded with splenocytes from immunocompetent mice, these cells displayed a high migratory ability toward the B16 chamber coupled to a slow B16 invasion extent. Conversely, IRF-8 deficient splenocytes displayed poor migratory extent toward the tumor, which reflected a high invasion ability of melanoma cells. This report constitutes the first evidence of mutual interaction between two different cell types in a microfluidic chip. Further analysis on time-lapse videos demonstrated that the migration of immune cells can be quantitatively and qualitatively evaluated by extrapolating single cell tracking profiles of each cell migrating toward melanoma cells (Agliari et al., 2014). From an oncoimmunology view, these tracking profiles represent relevant functional data expressing the overall behavior of immunocompetent *vs* non-immunocompetent cells faced to melanoma cells into this OOC system.

**FIGURE 1:**
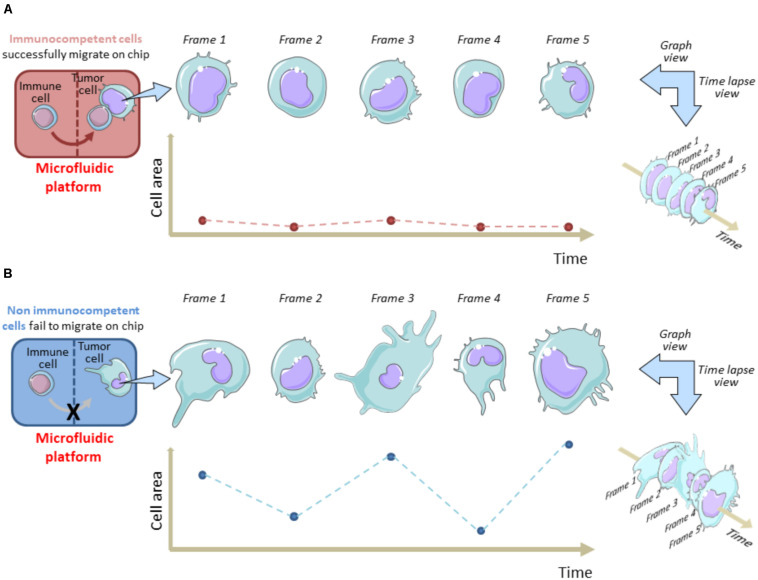
Tracking cell perturbation changes on chip during melanoma cell-immune cell interactions and after immune cell migration. A single cell area variation analysis is shown in immunocompetent **(A)** versus non-immunocompetent cells **(B)**. The area of a cancer cell is analyzed at fixed time intervals (e.g., 1 min) from an acquired time-lapse video, after the immune cell enters the cancer cell compartment and interacts **(A)** or fails to interact **(B)** with the malignant cell. Migrated immune cells do interact for longer time with cancer cells and this reflects in a minor frame-by-frame variation of the cell area. Hypothetically, this status is indicative of a dying/apoptotic cancer cell. This model underlines the migrate-to-interact strategy of an immune cell. The vertical brown/blue dotted lines within rectangles symbolically represent the complexity of the chip architecture and delimit the immune cell compartment from cancer cell compartment. The brown/blue dotted lines and points within graphs depict the area values for each frame referred to each time interval.

On the other hand, time-lapse microscopy is useful to evaluate the tumor cell membrane elasticity as a function of cell viability during their permanence in the OOC system. Fully functional tumor cells show high variations in the membrane fluctuation over each image frame (Figure 1). On the contrary, the membrane fluctuations are less evident in dying or stressed tumor cells. This perturbation factor has been quantified by Agliari and co-Workers by targeting time-lapse on single tumor cells inside the OOC. It can be used as a function of the stress status of a cancer cell after migration in the chip and reflects the ability of immune cells to limit cancer cell growth on chip as a consequence of tumor-immune cell interactions (Agliari et al., 2014). This “migrate-to-interact” concept can be adapted for every OOC platform designed to study how cancer cells respond to the presence of immune cells (Figure 1).

Further advancements in OOC development in oncoimmunology have been made by using human hepatitis B virus (HBV)-derived cancer cells and T cells that can be loaded and monitored on chip. Here, a customizable OOC was used to load engineered T cells expressing HBV-specific antibodies in liquid media with tumor cells derived from the same cancer patient. Tumor cells were loaded in a central chamber resuspended in a collagen matrix, thus recapitulating the TME on chip. In this setting, it was possible to follow and quantitate the tumor cell killing activity of T cells with accuracy (Pavesi et al., 2017). Moreover, this study allowed a better knowledge of the suitability of extracellular matrices for OOC systems, such as Matrigel, hydrogels or collagen complexes to better recapitulate the TME. Indeed, extracellular matrices represent a mix of extracellular proteins markedly improving the capability of cells to survive inside the microfluidic devices. In this situation, the cells engage chemical bonds with the components of these matrices then activating intracellular survival signals. Thus, the use of these matrices is particularly relevant for the activation and survival of some primary cell types, such as dendritic cells and hepatocytes (Mehta et al., 2010; Mölzer et al., 2019; Serna-Márquez et al., 2020). On the other hand, it is becoming increasingly evident that the use of gel matrices represents an important step to an optimal development of OOC platforms in oncoimmunology, as evidenced by the key role of the disparate types of matrices employed to reproduce the TME (Narkhede et al., 2017; Hassell et al., 2018; Sontheimer-Phelps et al., 2019).

In a recent study, Fang and co-Workers reported the realization of an OOC system with human tumor spheroids or murine organoids (Fang et al., 2019). They loaded the device with fibroblasts and MCF7 or 4T1.2 breast tumor cells to allow the on-chip generation of tumor organoids, with the fibroblasts spontaneously infiltrating the tumor mass. In this case they used the agarose as main biomaterial of the OOC. In addition, a microfluidic prototype has been developed by Borenstein and co-Workers to study the immune checkpoint inhibitors in human cancer (Beckwith et al., 2019). Specifically, this OOC system allows the trapping of a lung tumor biopsy and a real-time monitoring of the programmed cell death protein-1 (PD-1) expression. The originality of this study is represented by the development of an OOC platform optimized to monitor PD-1 on fresh biopsies in 24 h.

All these studies facilitated the *ad hoc* development of OOC systems and led to a new generation of more complex OOCs. Another representative example in using diverse cell populations in OOC research is the development of multi-organ units on chip, outlined by a recent work depicting a platform aimed at generating and connecting liver and gut on chip (Sung, 2020). The Supplementary Table 1 illustrates the technical and analytic details of several OOC systems and their potential use for oncoimmunology studies. This list suggests that OOC can be designed by lithographic techniques and 3D bioprinting methods, with or without gel matrices. As expected, the structural composition of the microdevices largely changes in function of their specific functional and analytic requirements.

## Analytic Tools to Study the Interactions Between Immune Cells and Cancer Cells in Microfluidic Platforms

Organs-On-Chip allow to study TME *in vitro* avoiding the use of expensive animal models in complex *in vivo* experiments. When planning an OOC experimental design, animal models should be used uniquely for initial validation of the specific OOC setting. Besides, OOC platforms are very useful for the extrapolation of hidden entities directly or indirectly associated to the migratory ability of immune cells and their capacity to reach and interact with cancer cells. These parameters are strictly dependent on the behavior of the two cell types within the OOC and, seemingly, in the TME. In this section we describe these hidden entities and their quantitation cues.

### OncoImmuno Chip: A Specific OOC System to Study Interactions Between Cancer Cells and Immune System

**FIGURE 2:**
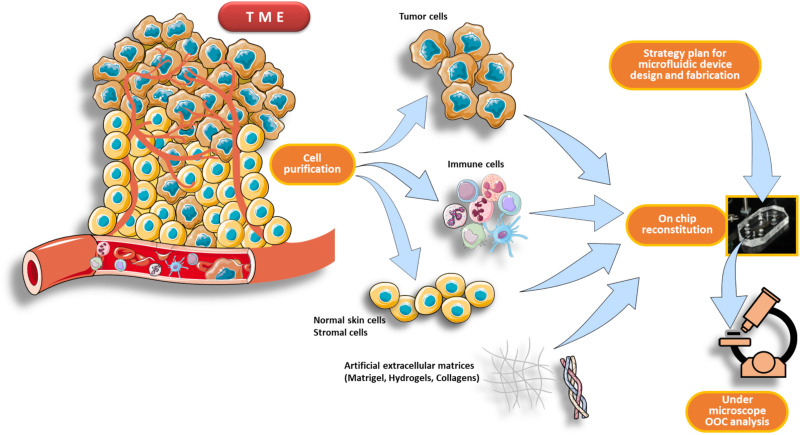
Schematic pipeline for TME reconstitution on an OncoImmuno chip. The first part of the workflow consists in a purification process of the main components of the TME, such as immune cells, tumor cells and normal skin cells plus stromal cells (if needed). When the design strategy of the microfluidic device is completed, the cells are then reconstituted inside the chip loading units. This reconstituted chip, resembling to an OOC, is then analyzed by a microscope platform. The TME can be derived from patients’ primary tumor or from an *in vivo* experimental tumor.

An *OncoImmuno chip* defines a microdevice in which the TME’s main components are isolated, reconstituted on-chip and coupled to time-lapse and image analysis systems to monitor immune and tumor cells overtime, for a maximum of 72 h (Biselli et al., 2017; Mencattini et al., 2020a; Torres-Simón et al., 2020; Figure 2).

**FIGURE 3:**
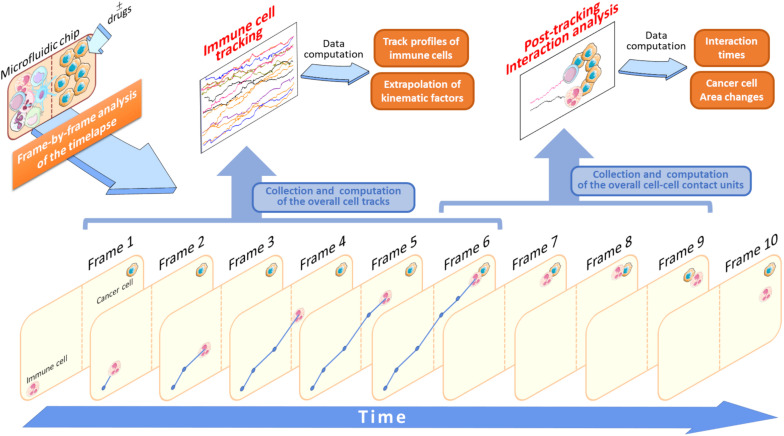
Application of the FBF tracking analysis to study immune cells versus cancer cells crosstalk. In this schematic model, the starting time-lapse recorded during the experiment execution is processed and all the variables associated to tracking analysis are extrapolated (Data computation). Specifically, when an immune cell is identified it will be tracked in each frame (Frame 1–6) to yield its associated trajectory (blue lines and points). This is done for each cell identified in the initial frame (Frame 1) of the video sequence. At the end of its walking, if the immune cell interacts with an adjacent cancer cell the duration of this interaction is computed and stored as a single interaction time value. In this example, this value is provided by the interval time difference between Frame 6 and Frame 9. The brown-dotted lines inside the microfluidic chip symbolize the compartment structures and ideally delimit the two immune and cancer cell compartments.

There are several important advantages to track immune cell migratory behavior toward cancer cells. For example, in an experiment where immune and drug pre-treated cancer cells are loaded into two separate compartments, it will be possible to acquire, frame-by-frame (FBF), a set of single cell tracks throughout the entire duration of the time-lapse (Figure 3). This FBF acquisition of immune cells yields an array of cell tracks, namely the *immune cell tracking profile*, whose behavior is strictly dependent on the anti-tumor effects of an administered drug (Figure 3). When each immune cell reaches the compartment containing drug-treated cancer cells, it interacts with them for a variable time. The computation of the duration of such an interaction is then fundamental for the quantification of therapeutic efficacy. Such a post-migration interaction represents a functional parameter indicative of the ability of the immune cell to “sense” cancer cells inside the device upon drug exposure (Figure 3).

**FIGURE 4:**
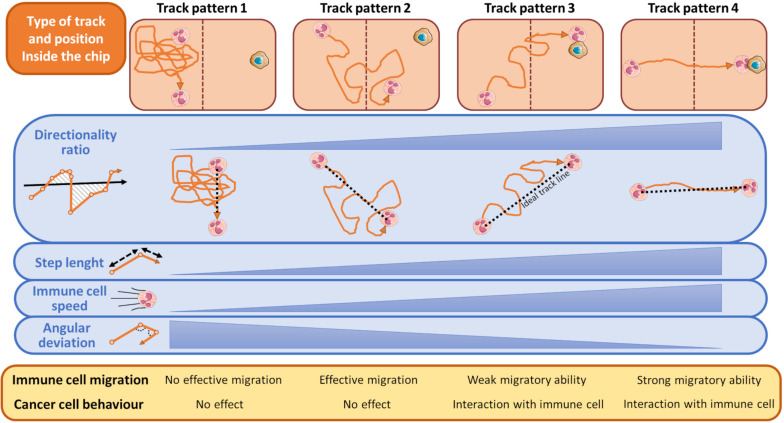
Schematic representation of most typical tracks of immune cells in relation to the presence of cancer cells during a time-lapse from an OOC experiment. During its walking toward the cancer cell, an immune cell can assume one of the indicated pattern motions. When the cancer cell exerts no influence on immune cells these can assume a track described by Pattern 1, namely a casual motion with no specific direction. This scenario is indicated by a very low directionality ratio compared to an ideal linear track (black dashed line), short mean step lengths, low speed, and high angular deviation of the immune cells. Conversely, if an immune cell moves forward by adopting the Track Pattern 4, it does successfully reach the opposite chip compartment and interacts with the neighboring cancer cell. This track is featured by an high directionality ratio (high linearity), step length and immune cells speed, and low angular deviation. In Track Pattern 2, immune cells pass through the opposite compartment but are unable to interact with the cancer cell. In Track Pattern 3 the scenario is similar to that described by Pattern 4, except for a low directionality ratio and then a weak migratory ability toward the cancer cell. Track Pattern 3 usually ends with a successful interaction between cancer and immune cell. The brown-dotted lines inside the microfluidic chip symbolize the compartment structures and ideally delimit the two immune and cancer cell compartments.

There are some typical classes of trajectories relative to immune cell motion that are highly indicative of the response to attractant or repellent substances emanated from tumor cells (Biselli et al., 2017; Comes et al., 2020b). These distinct track patterns have functional effect on cancer cell fate (Figure 4) and can be represented by several kinematic and non-kinematic factors. The first, *directionality ratio* (Gorelik and Gautreau, 2014), expresses the ability of an immune cell to reach its target cancer cell by taking the shortest route possible.

**FIGURE 5:**
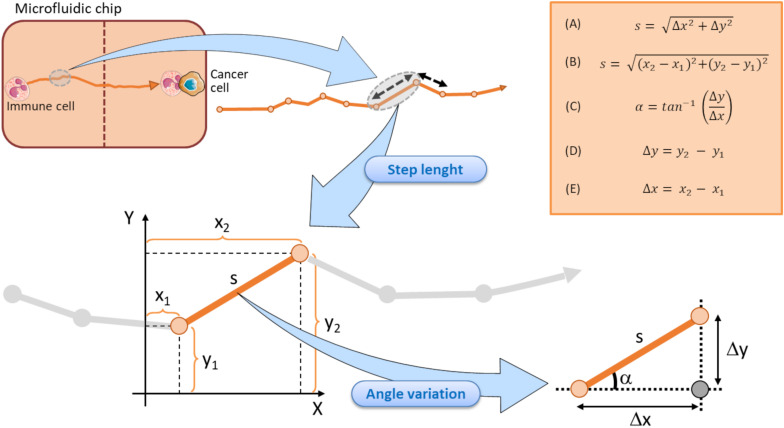
Schematic representation of the step length components in a tracking immune cell moving toward a cancer cell within a microfluidic chip. Each step length is calculated by returning the doublet of coordinates in starting (*x*_1_, *y*_1_) and finish (*x*_2_, *y*_2_) points. A single step length is depicted by the vector *s*, that can in turn be decomposed in the Δ*y* and Δ*x* units (representing the projection of the *s* vector to *Y* and *X* axes, respectively). Each step is associated to its relative angle deviation α conventionally referred to the *X* axis. Vertical dotted brown line symbolizes the structural components of the microfluidic device and ideally delimits the two immune and cancer compartments. Top right orange box displays the key formula associated to the step length and angle variation computations.

The *mean step length* of a track is another kinematic entity intrinsically associated to the track of an immune cell (Figure 4). It expresses the mean walking displacement of the immune cells between two consecutive points of the time-lapse (Figures 3, 4). Assuming that a single step length, *s*, is yielded by the formula A delineated in Figure 5 (Caplan, 1972; Simmons et al., 2010). Here, Δ*x* and Δ*y* represent the projections of the cell step length *s* on the *X* and *Y* axes in a Cartesian *XY* domain (Figure 5). By denoting as (*x*_1_, *y*_1_) and (*x*_2_, *y*_2_) the starting and the ending points of a generic displacement, i.e., △*x* = *x*_2_−*x*_1_ and △*y* = *y*_2_−*y*_1_, then the single step length of an immune cell can be also obtained by the formula B in Figure 5. The mean step length is calculated as the average *s* value among all the steps of the immune cell along its track and directly depends on the speed of that cell (Perner and Perner, 2009; Dewan et al., 2011; Biselli et al., 2017).

*Immune cell speed* returns the step length *s* from a starting (*x_1_, y_1_*) point to an ending point (*x_2_, y_2_*) divided by the time required to move from the two points (Figures 4, 5).

The track angle represents another important quantity of the immune cell migration path strictly associated to the directionality of the track. It is expressed by the angle *α* (in degrees) the current step vector *s* forms with the *X* reference axis (Figure 5). The *angular deviation* can be yielded by the difference between the two α values of the immune cell at two consecutive points.

Assuming that the step length is an *s* vector composed by its projection on *X* and *Y* axes, as mentioned in Figure 5, the angular deviation a constitutes the angle between the *s* vector and its projection on *X* axis. Such an angular deviation *α* of the immune cell can then be expressed with a generalized formula C in Figure 5, derived from the Pythagorean theorem, and is also a function of the attractive forces exerted by the adjacent cancer cell. As delineated by the two formula D and E in Figure 5, Δ*y* and Δ*x* represent the projections of the step lengths on *Y* and *X* axes, respectively. The two catheti Δ*y*, Δ*x* and the hypotenuse *s* constitute a right-angled triangle (Figure 5), where the angular variation α a is easily computable by the inverse tangent formula C in Figure 5 (Dummer et al., 2016; Lin, 2019; Cankaya et al., 2020; Zhang et al., 2020). Of note, each step length *s* of the immune cell track is indissolubly linked to its relative α value conventionally referred to the *X* axis, as evidenced in Figure 5.

Not less relevant, one of the most important parameter used to describe the type of motion for an immune cell is the *mean square displacement* (MSD)(Gorelik and Gautreau, 2014). Early studies that attempted to mathematically model cell migration in isotropic medium assumed that a cell moves like a Brownian particle (Dunn, 1983). Namely, an immune cell was assumed to undertake a persistent random walk, in which it moves directionally at short time intervals, but it loses its persistence at longer time intervals. The time to cross from the persistent regime to the random one is the directional persistence time, whose estimation requires a fitting of the MSD curve (Gorelik and Gautreau, 2014). The MSD is a crucial factor since it embeds global as well as local kinematic characteristics of the immune cell track at different time lag, thus evidencing cell behavior at shorter time but also the overall trend at larger time. In an oncoimmunology context, the MSD is strictly dependent on how cancer cell drug treatment can affect the immune cell trajectory on chip.

In conclusion, cell tracking analysis returns several hidden kinematic and non-kinematic values, that are an intrinsic feature of the FBF analysis in a time-lapse video. This can be easily extrapolated in an OncoImmuno chip, by analyzing the time-lapse video and single image frames with a specific image analysis software. In this regard, the open source software ImageJ is equipped with a number of plug-ins (i.e., Manual Tracking or Automated Tracking plug-ins) that allow to automatically process all the aforementioned entities, then facilitating the extrapolation of all kinematic factors associated to each single tracked immune cell. The use of ImageJ with the integrated Automated Tracking plug-in is a clear example of image-assisted analysis of cell migration tracking. A list of the most commonly used cell migration tracking software are depicted in Supplementary Table 2 (Emami et al., 2021).

Overall, the use of tracking analysis in OncoImmuno chips can recapitulate the interactions between cancer and immune cells in TME in the steady-state or following a defined therapy by exploiting hidden data acquired exclusively through these platforms and not accessible by *in vivo* TME methodologies.

### Generative Adversarial Networks to Evaluate the Interactions Between Immune Cells and Cancer Cells on Chip

The massive advent of sophisticated biotechnologies has been accompanied by a parallel evolution of instruments in which these technologies were computationally implemented. These analytical methods are recently exploiting the beginning of the Artificial Intelligence framework that is revolutionizing the field of biomedicine with its diverse applicative approaches (Ramesh et al., 2004; Buch et al., 2018; Balkanyi and Cornet, 2019; Holzinger et al., 2019; Mintz and Brodie, 2019; Kaul et al., 2020). In the area of machine learning (Rajkomar et al., 2019; Sidey-Gibbons and Sidey-Gibbons, 2019; Alsuliman et al., 2020), a novel kind of algorithm-based methodologies have recently gained a profound interest. These advanced methods belong to a sub-branch of machine learning, the deep-learning (Cao et al., 2018; Wainberg et al., 2018; Bock et al., 2021), and, in particular, often refer to the so called Generative Adversarial Networks (GAN). GANs can have disparate practices, covering social sciences, image analysis and biological problem solving. To this purpose, GANs have been applied for prediction of human actions (Liu et al., 2017), image processing applications (Nie et al., 2018; Li et al., 2019c; Xue et al., 2020; Yang et al., 2020), and computational analysis of natural environments (Saleemi et al., 2009; Negin and Brémond, 2019; Comes et al., 2020a, b). GAN are based on a game scenario where the generator network must compete with an adversary, the discriminator network. The two network models are trained together until the discriminator network is fooled about half the time from the image created by the generative network, meaning that it is generating plausible examples (Sahoo and Narayanan, 2019; Xu et al., 2020).

**FIGURE 6:**
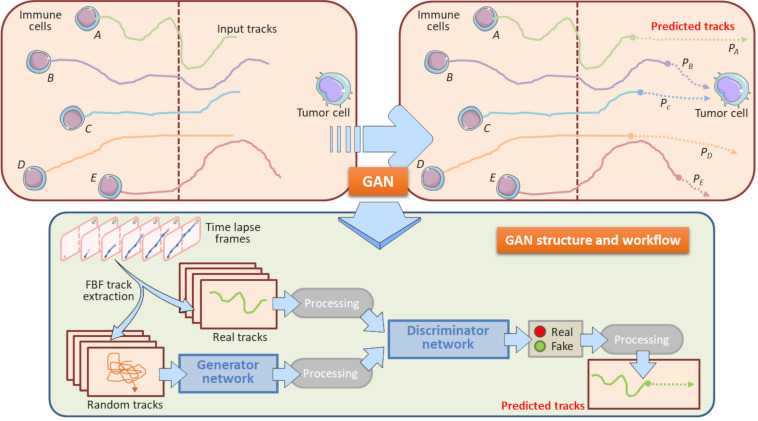
Role of GAN in immune cell trajectory prediction. Machine learning by GAN represents a practical manner to predict the whole track of a migrating immune cell. In this model, GAN predicts the migratory trajectory of immune cells (A, B, C, D, and E) and is helpful to foresee the behavior of immune cells when these encounter a tumor cell during their walking. GAN uses a Generator Network and a Discriminator Network to develop the predicted tracks from Real and Random tracks, extrapolated from each frame of the time-lapse. The Real tracks will then be processed into the Discriminator Network, whereas the Random tracks will constitute the input variables for the Generator Network, where they will be further processed and entered in the Discriminator Network. This Network exploits specific sigmoidal functions (Real, Fake) to distinguish the true predicted trajectories from fictitious tracks. The final result is constituted by all the predicted tracks associated to their input tracks originally entered. In this model, the computation of the predicted tracks P*_*B*_* and P*_*C*_* does allow to assert that the immune cells B and C will interact with the neighboring tumor cell with meaningful probability. Conversely, the predicted tracks P*_*A*_*, P*_*D*_*, and P*_*E*_* indicate that Immune cells A, B and E will interact with the same cancer cell with a very low probability. These information are relevant to check potential immune cells to be selected for post-migration Interaction time computations. Brown-dotted vertical lines symbolize the structural components of the chip and ideally delimit the immune and cancer compartments.

The gradually increased use of time-lapse microscopy together with the use of microfluidic platforms provided oncoimmunologists with potent tools to perform long-term live cell imaging and high-throughput analysis of on chip platforms (Comes et al., 2019, 2020a; Mencattini et al., 2020b). In this context, GANs constitute potent machine learning tools to predict how a cell migratory path will evolve starting from the real cell tracking captured by a time-lapse video. Specifically, an updated version of the GAN, social GAN (Comes et al., 2020b), can be useful to predict the post-migratory behavior of an immune cell when it is in proximity of a cancer cell in a microfluidic device (Figure 6). To this purpose, all the single immune cells are identified and tracked starting from the original time-lapse video sequence. To do so, GAN algorithm need to be “trained” by inputting a number of initial immune cell positions, namely the Input Tracks in Figure 6. After the training procedure, GAN is able to return high-fidelity immune cell trajectories by extrapolating them from GAN-dependent hidden entities (Fernando et al., 2019; Comes et al., 2020b). This approach is particularly useful to predict the behavior of poorly identifiable immune cells, or simply to accelerate the uptake of the experiment and to avoid phototoxic effects on the cells (Figure 6). GAN can provide a meaningful estimation of the probability for each immune cell to physically interact with the cancer cell in the vicinity (Figure 6).

In conclusion, a GAN learning system can be helpful for the envision of the so called *in silico* OOC experiments (Swayden et al., 2019). The main goal of such a system is the evaluation of a real OOC experiment without performing until the end reducing time and experimental costs. Moreover, this process can also generate an *in silico* OOC simulation potentially useful to plan new optimized OOC experiments.

## OOC Systems for Drug Investigations in Oncoimmunology

One of the most active research fields in oncoimmunology is the dissection of drug properties for cancer therapy. OCC platforms represent a valid alternative in this context. Indeed, in the last years OOC systems have shown potential for discovering indirect or direct drug properties on tumor cells. Simple OOC systems have been developed in which only target cells and drugs are loaded to assess direct effects of the drug (Kwak et al., 2014; Shin et al., 2016). In this section we will review the impact of microfluidic devices in boosting research on oncoimmunology in the context of anti-cancer therapy, providing an innovative tool to evaluate how drugs can affect the interactions between cancer cells and immune system.

### OOC Systems for the Evaluation of Anti-Tumor Therapies

One of the first studies aimed to assess the effects of pharmacological treatments for cancer therapy by using an on chip approach is represented by a study addressing the role of formyl peptide receptor 1 (FPR1)/annexin a1 (Anxa1) axis in anti-tumor response to anthracycline-based chemotherapy (Vacchelli et al., 2015). In this report, OOC experiments in humans and mouse settings were crucial to demonstrate the importance of the integrity of the FPR1/Anxa1 axis to allow immune cell migration and interaction with cancer cells undergoing chemotherapy-induced immunogenic cell death. Indeed, cancer cells treated with anthracyclines (i.e., Doxorubicin or Mitoxantrone) release Anxa1, which acts as a danger signal for immune cells. Single cell tracking profiles extrapolated by OOC system displayed that FPR1 expressed by immune cells, particularly dendritic cells (DCs), is required to “sense” Anxa1 emanated from dying tumor cells and to migrate toward them engaging in stable interactions. Interestingly, in the OOC setting only DC with an intact FPR1 successfully migrate toward anthracycline-killed tumor cells and capture apoptotic bodies released from these dying cells. Post-analysis of time-lapse video recordings in this OOC system allowed the extrapolation of high numbers of single cell tracks associated to high interaction times between immune cells (including DCs) and cancer cells, showing that immune cells with intact FPR1 perform biased random walks toward anthracycline-treated cancer cells, while those with mutated FPR1 show uncorrelated random walks (Biselli et al., 2017). Thus, numerical descriptors and statistical physics applied to OOC systems provide an additional tool for a quantitative description of the immune response to cancer during chemotherapy.

Organs-On-Chip systems can undergo further step-by-step implementations in their structural complexity which do not compromise their state-of-simplicity. In this regard, Nguyen and co-Workers have successfully visualized and quantified the effects of the anticancer drug Trastuzumab in a TME reconstituted on chip, composed by cancer-associated fibroblasts (CAF), an HER2^+^ breast cancer cell line (bearing a HER2-receptor gene amplification), and immune cells (Nguyen et al., 2018). This study demonstrates that an OOC system hold potential for further implementation by adding other TME key cell components, such as cancer associated fibroblasts (CAFs). Addition of CAFs in this system still contributes to maintain a simplified and smart OOC platform. Here, the OOC system has been utilized to dissect the role of Trastuzumab, a reference anti-cancer for HER2+ breast cancer patients, on such a tumor-on-chip complemented with CAFs plus hydrogels to reproduce a central endothelium compartment. The microdevice is constituted by two side chambers, containing the immune cells (embedded in a collagen matrix), cancer cells and CAFs, and a central compartment with endothelial cells. These chips where monitored with or without the presence of Trastuzumab. The extrapolation of interaction times, cancer cell speed and tracking profiles by time-lapse demonstrated that Trastuzumab inhibited breast cancer cell growth with a concomitant induction of cell death. In addition, CAFs hindered the interactions between cancer and immune cells. Overall, these on-chip observations suggests that this tumor-on-chip effectively recapitulates the *in vivo* scenario referred to the effects of Trastuzumab in human breast cancer. A similar concept was applied in a study illustrating the crosstalk between lung cancer cells and epithelial cells in an OOC system mimicking the lung alveoli. Here, H1975 non-small-cell lung cancer (NSCLC) cells were loaded in a microfluidic device to evaluate the effects of Erlotinib and Rociletinib, two representative third generation Tyrosine Kinase inhibitors. The authors designed a chip composed by two side compressible chambers for air flow, mimicking respiratory processes, and a central compartment further subdivided in two separated chambers, containing cancer cells and endothelial cells. This design had the purpose to recapitulate the alveoli of lungs, under normal or cancer settings. These studies not only showed the feasibility of this lung-on-chip system to study the anti-tumoral effects of Tyrosine Kinase inhibitors, but also revealed an unexpected role of breathing in contrasting cancer growth (Hassell et al., 2018).

Organs-On-Chip systems have also been exploited to investigate the role of epithelial-mesenchymal transition (EMT). For instance, an interesting OOC model has been generated to study these phenomena. Here, EMT has been recreated on chip with human lung tumor cell spheroids (NCI-H460 or HCC827 cells) resuspended in Matrigel and human umbilical vein endothelial cells (HUVECs). The microdevice is composed of a nylon mesh matrix which recapitulates the separation system between cancer cells and HUVECs. In this microdevice tumor cells and HUVECs are separated by the mesh membrane. When recombinant TGF-β was added in this OOC system tumor spheroids displayed an activated expression of the EMT factors Snail, and Akt, paralleled by decreased levels of E-cadherin. In addition, tumor cells acquired the ability to migrate toward HUVECs. This OOC system may represent an optimal model to study the effects of anti-cancer drugs in terms of control of processes involved in EMT (Li et al., 2019a). Another representative example of on chip anticancer agent evaluation is the development of a metastasis-on-chip model of study (Wang et al., 2020). This OOC is assembled with methyl methacrylate for structural and spatial components, whereas PDMS is used to allow gas and liquid exchange between cells. The PDMS is used to create sheets to which a 10 μm sized resin membrane is sliced. This PDMS plus resin system is then inserted inside the OOC and used for cell loading. Such device was employed for the evaluation of anti-tumor activity of 5-Fluorouracyl (5-FU) on Caki-1 kidney cancer cells loaded with immortalized HepLL hepatocytes. Of note, this OOC system was integrated with a programmable electronic pump to feed cells with nutrients in the chip. This study can inspire the generation of implemented metastasis-on-chip platforms suitable for other metastatic tumors.

The described OOC platforms represent a starting point for implemented systems specially designed to evaluate the efficacy of emerging immunotherapy strategies in the presence of immune cells. Recently, OOC devices were successfully employed to culture murine- and patient-derived organotypic tumor spheroids preserving the immune compartment of the TME and to evaluate the sensitivity and resistance to PD-1 blockade (Jenkins et al., 2018). This system accurately recapitulated the *in vivo* scenario, was compatible for long-term culture (up to 1 month) and allowed to evaluate multiple parameters of response, such as cell death and growth of tumor organoids from mice or patient-derived organoids loaded inside the microdevice. The microdevice consists in two side chambers utilized to load the conditioned medium (i.e., the anti-PD-1 antibodies) and a central compartment employed to load human tumor spheroids or tumor cells from mouse colon cancer lesions which develop organoids several days after loading (Aref et al., 2018). This platform can be adapted to evaluate other immune checkpoints (i.e., CTLA-4) expressed on cancer cells. OOCs have also revealed as useful systems to study the effect of folic acid in ovarian cancer. Folic acid is the elected anti-tumoral drug to cure this and other solid malignancies. Here, Wimalachandra and co-Workers have proposed an OOC in which the main chamber was rounded by HUVEC cells and filled with OVCAR-3 tumor cells. This microfluidic device has been used to evaluate the migration of DCs and T cells, in presence or absence of folic acid-loaded nanoparticles (Wimalachandra et al., 2019). These encouraging studies suggest not only that nanoparticles are a useful to assess anti-cancer drugs on chip, but can also inspire the use of OOC platforms for the study of tumor cell functions by nanoparticle-encapsulating drugs.

### OOC Applications for the Evaluation of Anticancer Drug Combinations

Single agent-based strategies are often insufficient for a successful and complete tumor eradication in cancer patients. Current strategies aim at combining therapies with a second (or even third) drug to amplify anti-tumor responses and/or to broaden the spectrum of responding patients (Bao et al., 2020; Crunkhorn, 2020; GajdÁcs et al., 2020; Iratni and Ayoub, 2020; Jonnalagadda et al., 2020; Karimi et al., 2020; Kawachi et al., 2020; Kim et al., 2020; Mollica et al., 2020; Qian et al., 2020; Song et al., 2020; Yamashita et al., 2020; Zhu et al., 2020). The development of microfluidic devices for oncoimmunology applications based on the use of drug combinations is only beginning to emerge. One of the major challenges to deal with is the precise architectural optimization of the chip devoted to these purposes. Indeed, the evaluation of multiple factors on a single chip implies an adjunctive difficult level, not only in the chip fabrication, but also in choosing the bio-architectural components of the chip. For instance, if one plans to construct a complex 3D chip this should not compromise the control easiness of the cells within it. In this regard, an example comes from an elegant report where Caco-2 colorectal adenocarcinoma cell lines were co-cultured on an OOC platform with hepatic HepG2 cells acting as an artificial liver producing metabolites. This microfluidic system has been successfully employed to evaluate and quantitate the effects of Irinotecan and Temozolomide, alone or in combination (Jie et al., 2017). Noticeably, a superior ability of the double condition to induce apoptotic cell death of tumor cells has been showed. This encouraging study suggests that a chip-based system, when properly designed and loaded with the right cell subsets can be effectively exploited to follow unexpected drug combination effects. Other similar findings come from a simple CC system optimized for use of two anticancer agents at different drug concentrations. Here, PC3 tumor cells have been loaded with Doxorubicin or Mitoxantrone with TRAIL (Kim et al., 2012; Desyatnik et al., 2019).

**FIGURE 7:**
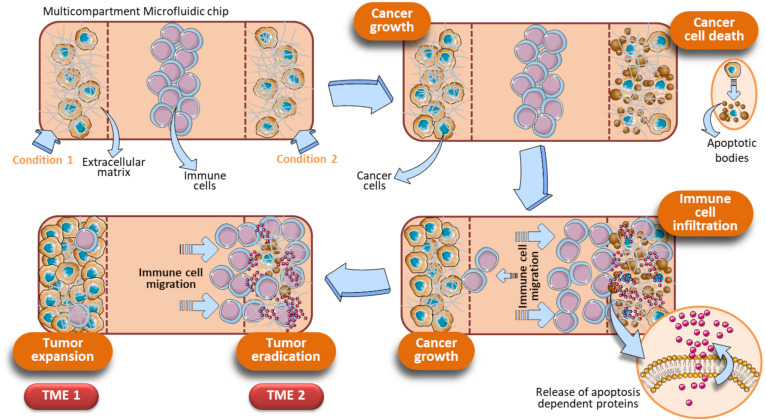
Schematic representation of a dual condition chip to study the dynamics of apoptosis-induced anticancer drugs. Immune cells are loaded in the central chamber of the device. In parallel, cancer cells were loaded in presence of an extracellular matrix (e.g., Hydrogels, Collagen, or Matrigel) and specific drugs to be evaluated (Condition 1, Condition 2). Assuming that the Condition 1 is represented by untreated cancer cells and Condition 2 by tumor cells exposed to a cell death inducer (or a drug combo whose final action is cell death), these different on chip parallel treatment trigger a gradually divergent behavior of the cancer cells, reflecting in the final generation of two different TMEs (TME 1 and TME 2). TME 1 generates when the Condition 1 is a pro-tumoral drug or no treatment. In these cases, cancer cells will proliferate and a growing tumor will develop (TME 1). Concomitantly, an opposite effect due to tumor cells treated with anticancer agents occurs in the opposite microfluidic chamber (TME 2). The TME 2 is shaped by immune cells, that migrate under the effect of apoptotic factors released by cancer cells upon treatment with anticancer drugs. How immune cells shape the TME development is strictly dependent on how cancer cells are treated in the two side compartments. The initial key event of the TME 2 is the generation of apoptotic bodies as a consequence of tumor cell killing operated by immune cells. When cancer cells are exposed to a drug combination (Condition 2), the associated Condition 1 at the opposite side chamber can be a single drug of that combination or no exposure. Vertical dotted brown lines symbolically represent the structural units of the device.

A significant step forward is the optimization of OOC for evaluating drug combinations by taking into account the role of immune cells to provide a more complete and faithful scenario. OOC platforms have been developed employing immune cells *in toto* and cancer cells resuspended in Matrigel adapted for the exposure to two different drugs acting alone or in synergy. This OOC design has allowed to demonstrate the superior anticancer activity of the DNA demethylating agent Decitabine (DAC) when administered with Interferon-α (IFN-α) against melanoma in a competitive assay (Lucarini et al., 2017). In detail, this device is composed by a central chamber used to load immune cells (PBMC from healthy donors) and two side channels for loading Matrigel-resuspended human A375 metastatic melanoma cells with or without DAC and IFN-α, alone or in combination. Fluorescence microscopy evaluation and time-lapse experiments have shown that immune cells preferentially migrate toward the A375 cells containing both drugs rather than each single agent (DAC or IFN-α), or no drugs. This approached mirrored *in vivo* studies performed in melanoma-bearing mice, where combined DAC and IFN treatment promoted immune cell infiltration, limiting tumor progression (Lucarini et al., 2017). The same OOC device was employed to evaluate the behavior of specific immune cell subsets, i.e., dendritic cells (DC) toward tumor cells undergoing combined therapy. Here, IFN-α-conditioned DC (IFN-DC) were monitored within 3D tumor spaces for their ability to infiltrate and engulf collagen-embedded SW620 colorectal cancer cells treated with a combination of drugs (Romidepsin and IFN-α) with respect to untreated (Parlato et al., 2017). We hypothesize an OOC scenario in which DAC induces cancer cells to undergo apoptosis, with a concomitant release of apoptosis-dependent chemokines or *via* the activation of pathways strictly related to cell death such as the FPR1/Anxa1 axis (Vacchelli et al., 2015). In parallel, IFN activates immune cells as they infiltrate the matrigel microenvironment. This, in turn, favors immune cells to interact with A375 cells and to complete their tumor cell killing activity, leading to further release of apoptotic factors. On the other hand, in the opposite side chamber containing the single drug or saline, immune cells fail to infiltrate the melanoma microenvironment. Overall, this system can be regarded as an OOC provided with two functional TMEs, each responding to local environmental stimuli represented by direct effects of the drugs and by subsequent infiltration of immune cells (Figure 7). Of note, this dual competitive condition chip can is an useful tool to evaluate how these differently shaping TMEs will evolve in function of the immune cells behavior. Indeed, the preferential migration of the immune cells toward one of the two conditions is the peculiar effect noted in this particular device, and is strictly dependent on type of drugs cancer cells are exposed to. To this extent, tumor cells can be elegantly monitored by using this specific dual condition chip to study the cellular dynamics of some drugs endowed with cell death-induction activity, compared to an internal control (Figure 7).

## Microfluidic Devices for Studying the Effects of Radiations in Immune Cells and Cancer Cells

Radiation therapy (RT) is employed to cure localized cancers and is known to act both by killing malignant cells and by activating anti-tumor immune responses (Atwell et al., 2019, 2020; Bellia et al., 2019; Fellin et al., 2019; Chen Y. et al., 2020; Gupta and Chatterjee, 2020; Koukourakis and Giatromanolaki, 2020). Despite the wide use of RT to overcome the main problem of cancer resistance, the mechanisms by which radiations activate immune system to kill cancer cells are still under intense debate. Tumor mouse models constitute an invaluable tool to carry out research on how immune system interacts with cancer cells in the TME under RT regimens (Frey et al., 2012; Thiemann et al., 2012; Du et al., 2016; Dobiasch et al., 2017; Keisari, 2017; Bellia et al., 2019; Domankevich et al., 2020; Hader et al., 2020; Qin et al., 2020). The exploitation of animal models in this context has played a relevant role for the determination of radiation doses to be used to adequately activate immune cells, or certain type of immune cells, to efficiently eradicate cancer. These premises clearly evidence that RT represent an important strategy to fight cancer and hence is of key relevance for oncoimmunology research. Considering the evident limitations in animal handling imposed by European community legislation (Bassi et al., 2020; Mocho, 2020; Tian et al., 2020; Vinken, 2020), the generation of innovative tools to investigate how radiations can affect the immune system in fighting cancer represent an urgent need.

Recently, microfluidic systems have been adapted to investigate the anti-cancer effects of RT. An example of this type of approach has been carried out for an on-chip study of RT in head and neck squamous cell carcinoma (HNSCC) (Cheah et al., 2017). A very simple PDMS-based OOC system has been designed to investigate how single dose irradiation (5, 10, 15, and 20 Gy) could affect the growth of HNSCC primary tumor cells derived from patient biopsies. This OOC is composed by a central chamber connected to an inlet and an outlet well. The wells are connected to the chamber by a single microchannel. The chamber has been used to insert a little fragment of patient’s biopsy, and the inlet well serves to let media to flow-through in the OOC system. Irradiation of HNSCC tissue in the microfluidic device was carried out inside a custom-made Perspex Block (Butson et al., 1996), adequately applied to the microfluidic device. The irradiation doses of the tumor tissue were chosen as appropriate as to avoid the degradation of the microdevice biomaterials. This device allowed to follow the effects of RT on HNSCC tissues derived from primary tumors or metastatic lymph nodes. This study represents an interesting attempt to validate the concept of personalized medicine by using an OOC platform coupled to RT systems.

Lately, a new CC has been developed to study effects of radiations on soft tissue sarcoma (STS) (Patra et al., 2019). This device has been employed to follow the effect of combined RT and chemotherapy (CT) on STS spheroids grown inside the chip. Three different radiation doses were used (0.5, 2, and 8 Gy), combined with Doxorubicin at two different concentrations (2 and 20 μM). The device has been designed to favor the isolation of growing spheroids after the tests. This allowed to further analyze the spheres by flow cytometry (to evaluate apoptosis) and clonogenic assays (to evaluate survival). Spheroids were also monitored for changes in the size as a function of tumor progression. Two different tumor cell lines, STS93 and STS117 were used. The STS117 cells, bearing a mutated p53 protein, appeared to be more resistant to the combined action of Doxorubicin plus RT, compared to the STS93 tumor cells (bearing the WT p53). The device is constituted of a 5 × 3 array chamber, each containing 24 microchambers for STS spheroid trapping and culture. This chip was used to appropriately apply two independent RT and CT gradients around the growing spheroids. The irradiation apparatus is constituted by a clinical linear accelerator system that does not affect the microdevice biomaterial quality (PDMS). This representative study proposes a simple method to generate RT and CT combination gradients on chip to apply different radiation doses. In a similar work based on the use of ovarian cancer cell lines OV1946, a microfluidic cell culture platform equipped with a spheroid culture chamber array was designed. Although this study is not directly employed to test cancer drugs, the authors propose it to be used in combination with drugs and RT because of the peculiar configuration of the PDMS chip. The chip is composed by a 4 × 4 array of main chambers, separated by magnetic-actionable sensors (700 μm metal bar, in opened/closed states) which redirect the liquid flows through the chambers. Each chamber contains a 5 × 5 matrix of spheroid-trapping microwells (300 μm diameter), to yield a maximum of 25 replicates per chamber. In this system, OV1946 cells were left to generate spheroids in microwells and then RT (8 Gy) is given to appropriate chamber by a GammaCell3000 system. The presence of the actionable sensors allows to combine RT with a drug flowing into the desired wells. This system allows tumor spheroids to be monitored overtime upon RT and CT, with different radiation doses and drug concentrations (Brunet et al., 2017).

To date, no studies have been performed yet to investigate how radiation can impact on the crosstalk between immune cells and cancer cells on chip. Considering that RT often exerts immunogenic effect on cancer cells, a dual tumor-immune OOC may represent a valid alternative to study the behavior of immune cells in presence of RT-treated tumor cells (Figure 7).

## Use of OOC Platforms to Study Patient-Derived Xenografts

The use of laboratory animals has been widely exploited in studies involving microfluidic chips, due to the availability of immune cells from representative organs composing the immune system (i.e., bone marrow, spleen, and lymph nodes)(Mattei et al., 2010; Hickey and Chow, 2017; Elmore, 2018). On the other hand, the demand of immune cells still represents a great obstacle when planning microfluidic experiments with human cells. In fact, blood circulating immune cells can be easily available but cells residing in bone marrow, spleen and other lymphoid organs are unreachable. Based on these premises, there is an urgent need to overcome these difficulties to allow microfluidic experiments to be carried out in translational and clinical research contexts.

Recently, innovative humanized models have been developed for cancer immunotherapy research (Sanmamed et al., 2016). Among these, patient-derived xenografts (PDX) mice are immunodeficient animals adapted to receive a fragment of a fresh tumor excised from a cancer patient (Cho, 2020; Powley et al., 2020). A major advantage of PDX models, compared to simple cell line transplants, organoid xenografts or traditional tumor mouse models is represented by the retention of the TME architecture and genetic complexity of patient’s tumor. In this manner, the human tumor can be faithfully recapitulated in PDX mice.

**FIGURE 8:**
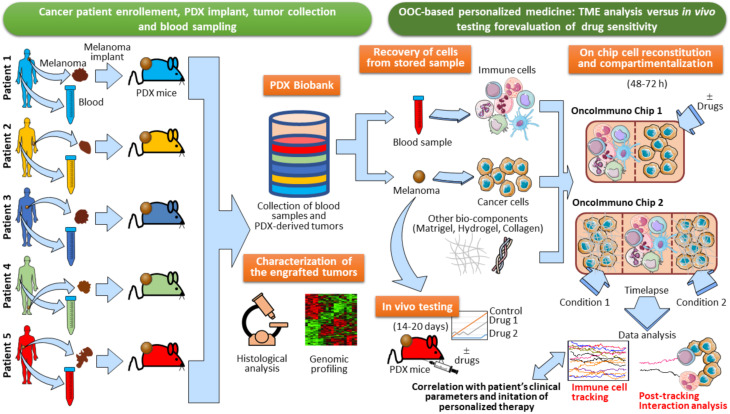
Establishment of PDX-based OncoImmuno chip platform for melanoma patient’s personalized therapy. Melanoma lesions and blood samples are collected from enrolled patients. and tumors are then implanted in PDX mice. When the tumors reach an adequate size, they are excised and stored in a Biobank with the associated blood samples. In parallel, samples are assayed for immunohistochemistry, Hematoxylin/Eosin staining and Gene expression profiling. When a patient is recruited to initiate a therapy, there are two alternatives to assay the sensitivity of anticancer drugs: *in vivo* testing with re-engraftment of the stored melanoma lesion (requiring 14–20 days), and OOC-based analysis (requiring 48–72 h). Immune and cancer cells are obtained from the patient’s blood and tumor tissue, previously stored in the Biobank. Cells are then loaded in several types of customized microfluidic chips, with simple (OncoImmuno chip 1) or complex compartmentalization levels (OncoImmuno chip 2). Complex compartmentalization may require the use of other bio-components mimicking the TME matrix and the stroma (Matrigel, Hydrogel and collagen). In OncoImmuno chip 1, immune cells are left untreated whereas cancer cells are exposed on chip to a drug or left untreated (control condition). In OncoImmuno chip 2, Immune cells can be left untreated or treated with a single drug (Condition 1), whereas cancer cells on the opposite side chamber are exposed to a drug combo (Condition 2). Time-lapse is then started and the resulting video sequence is used for automated single cell tracking. Drug sensitivity is assayed by the extent of migration of immune cells (Immune cell tracking). To assess the vitality of melanoma cells, FBF area variation of these cells is evaluated and interaction times between cancer and immune cells are assessed (Post-tracking interaction analysis). For OncoImmuno chip 2 preferential migration of immune cells toward Condition 1 or Condition 2 is determined. Tracking profiles, interaction times and the associated kinematic variables will be compared to patient’s clinical parameters and exploited to start the cancer patient’s personalized therapy. Vertical brown dotted lines symbolically depict microfluidic device structural units and ideally delimit the immune and cancer compartments.

In perspective, PDX models may represent an invaluable tool for oncoimmunology studies of human tissues by microfluidic systems. Indeed, one can collect blood samples and tumor lesions from cancer patients in order to generate a biobank of patients’ specimens. Little fragments of the tumor lesion excised from each patient can be transplanted in PDX mice (Figure 8). The resulting growing tumor in PDX mice is characterized for genomic profiling and histological analysis to confirm the TME morphology and TME architecture fidelity (Figure 8). At this point, *in vivo* drug testing can be performed in the PDX animals. This will allow to determine the therapeutic efficacy of anticancer agents or combination of drugs. The limitation of this approach, however, is the impossibility to evaluate immune responses, due to the fact that the PDX mice display an immunocompromised compartment. In this respect, OOC platforms may be used as an extension of PDX models. Indeed, autologous immune cells from patient’s blood sample can be loaded onto the chip together with a fragment of the tumor lesion from the same patient. The use of devices with different compartmentalization levels (e.g., Microfluidic OncoImmuno Chip 1 and Microfluidic OncoImmuno Chip 2, Figure 8) allows more reliable cell tracking results and may constitute a doubly mutual control. In addition, multi-compartment chip systems open new alternatives in the evaluation of a single drug or drug combos. Interestingly, these PDX-based OOC systems may represent a smart and reliable approach to study the crosstalk between cancer cells and immune cells all derived from a specific cancer patient (Figure 8). Noticeably, the development of PDX-based OOC platforms is a valid alternative to the expensive *in vivo* drug evaluation and permit to obtain the results in quick time, compared to the *in vivo* drug testing, requiring 14–20 days for results (Figure 8). In summary, PDX-based OOC systems can be viewed as an added value to *in vivo* humanized models and a potential tool for personalized anti-cancer therapies.

## The Human-On-Chip: a New Frontier in Oncoimmunology?

Organs-On-Chip platforms currently used to investigate the disparate open questions on oncoimmunology are gradually evolving in complex systems formed by multiple interconnected OOC units. The main purpose of such an intricate OOC network will be to study specific immune organ’s physiology in different types of cancer. This innovative approach certainly constitutes a future useful application for oncoimmunologists. In addition, it can represent the best platform ever developed, recapitulating the *in vivo* scenarios with superior affordability. However, the lack of definitive standardization criteria and the persistence of poor ethical policies for applications of advanced OOC systems in clinical research represent a strong limitation to this evolution.

**FIGURE 9:**
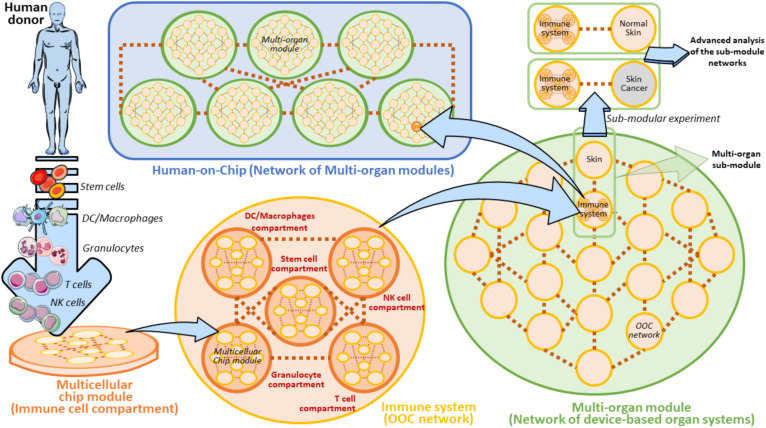
Modular view of a Human-on-Chip with emphasis on a hypothetical on chip immune system model. A Multicellular chip module is a unit containing all the key cellular components and extracellular factors derived from a specific human donor’s immune cells. In the Immune system these organs can be represented by five main cell populations (Stem cells, DC/Macrophages, Granulocytes, T cells, and NK cells). These cell subsets are properly loaded to form the five equivalent Multicellular chip modules (Immune cell compartments). Several Multicellular chip modules can generate an OOC Network. In this modular view, Immune system can be defined as an OOC Network composed by five Multicellular chip modules. Several OOC Networks compose a superior network of device-based organ systems, namely the Multi-organ module. The Human-on-Chip is composed of several serially connected Multi-organ modules. A Multi-organ module can also contain several subunits (Multi-organ sub-modules) to be employed for some isolated and targeted experiments (Sub-modular experiment) in which the sub-module is separated by its own Multi-organ module. In the case of the Immune system on chip, a Sub-modular experiment may be planned by a comparison of two connected OOC networks derived from two Multi-organ sub-modules (Immune system versus Normal skin or Skin cancer, respectively). Such an experiment can be aimed at comparing the immunosurveillance network activity to normal skin versus skin cancer. These experiments can be carried out with the help of AI algorithms and system biology approaches. Brown dots ideally represent the interconnections between the depicted units. The light-blue arrows evidence the passage to gradually superior levels of module complexity.

Whether or not the development of a Human-on-Chip (HOC) will symbolize a groundbreaking frontier in oncoimmunology is still an unsolved question. The generation of HOC platforms is a common ambitious objective of several ongoing multidisciplinary projects. Besides, microfluidic platforms are beginning to be designed having in mind the requirement of multiple cell types and multi-compartmentalization approaches (Zhang et al., 2009; Berry et al., 2020; Langerak et al., 2020; Sung, 2020). However, the road to obtain a fully functional HOC platform is still far to be reached and certainly fraught with obstacles. The final purpose of the HOC platform development must necessarily include the connection of at minimum two multicellular chips to recapitulate the *in vivo* function of at minimum two equivalent organs (Vunjak-Novakovic et al., 2013; Skardal et al., 2016; Sontheimer-Phelps et al., 2019). This implies that a HOC platform will be effectively finalized when at least a set of key human organs will be successfully connected in a complex OOC network, accompanied by AI (Artificial Intelligence) analysis algorithms, i.e., GAN (Comes et al., 2020a; Mencattini et al., 2020b), and advanced system biology tools (Jurisic, 2020). Such an intricated HOC can be conceived as a modular network where every single node is represented by a matrix of properly interconnected multicellular chip modules. Each Multicellular chip module recapitulates a physiologically functional human cell compartment on a microfluidic device (Figure 9). There are disparate obstacles to the composition of this modular HOC. One of the most relevant difficulty comes from the identification of the precise way each multicellular chip module must be interconnected with the units in close vicinity. Another problem can be to establish how these OOC should communicate with distant modules. Hence, a solution to overcome these issues may be the development of a complex module-based microfluidic network representing modular structures composed of interconnected units (Figure 9). In this scenario, a fully modular HOC model can be theoretically hypothesized, formed by Multicellular modules, OOC networks and Multi-organ modules, accurately interconnected to compose a functional HOC network recapitulating human physiology (Figure 9). Of note, each module unit is always constituted by inlet and outlet connections, denoted by the two points of each connection line between units. This will theoretically sustain fluidic circulation in the system.

It is widely reported that bone-marrow, lymph nodes and spleen represent key immune organs maintaining immune cell homeostasis and immune surveillance (Le Bouteiller and Asherson, 1982; Olszewski, 2003; Mattei et al., 2010; Snell et al., 2012; Ruddle, 2016). In our modular HOC model, the immune system is defined as an OOC network of several main immune cell compartments derived from a human donor (Figure 9). These compartments are defined as heterogeneous containers of a certain cell subtype. For example, DC/Macrophages compartment is represented by all the common types of dendritic cells existing in the immune system (i.e., Conventional DCs, Plasmacytoid DCs, etc.) (Austyn, 2016; Tucci et al., 2019). When another OOC network (e.g., a skin OOC network) is directly connected to the OOC immune system we obtain a Multi-organ sub-module defined by two unique OOC networks. Interestingly, this sub-module can be easily re-developed as an isolated entity to perform specific sub-modular experiments (Figure 9). These targeted experimental layouts are oriented at extrapolating the complex crosstalk between the immune system and skin on chip, and represent an important starting point to the development of a module-based HOC platform (Henderson et al., 2020). Specifically, normal skin could be substituted by skin cancer cells (e.g., KRT14 primary skin cells and A375 melanoma cells) being expressed by circulating tumor cells and metastases (Lima et al., 2017; Hanley et al., 2020; Ibrahim et al., 2020), in order to carry out comparative sub-modular strategies between two different sub-module units. These units uniquely differ for the presence of normal skin cells or melanoma cells. In these settings, it is possible to compare how the immune system does respond to the presence of normal skin cells or melanoma in confined sub-modular microfluidic experiments (Figure 9). This theoretic modular model can be a good starting point to plan the development of distinct on chip organ systems defining a specific function (or set of functions) in the human body.

Recently, several bodies of evidence have been published proposing complex and functional HOC, which have been developed by exploiting the modular concept and submodular experiments illustrated in Figure 9 (Vunjak-Novakovic et al., 2013; Skardal et al., 2016; Sontheimer-Phelps et al., 2019). Vunjak and co-Workers finalized a first validated Heart-Liver-Vascular HOC prototype, experimentally suitable for drug testing (Vunjak-Novakovic et al., 2013). These studies inspired the creation of a HOC platform based on modular organoid-containing microdevices. Here, the authors proposed the setting up of a complete circuitry to recapitulate a modular microfluidic system where each organoid-based OOC is adequately connected to form a HOC system composed by three modular liver, cardiac, and vascular organoid-containing microdevices. This platform is well suitable to study the effects of drugs in hepatic tumors, i.e., by evaluating the organoid sizes, immune cell infiltration or drug toxicity in each microdevice. This device was recently employed to quantitatively evaluate the effects of Propranolol and Epinephrine on this multiorgan platform (Skardal et al., 2016). These recent efforts represent an encouraging promise to an affordable and safe engagement of HOC for oncoimmunology purposes.

## Discussion and Conclusion

The advent of microfluidic technology is gradually impacting the field of biophysics and biomedicine. Microfluidic devices are smart systems potentially useful in disparate and divergent types of scientific problems covering medicine, including research in tumor immunology.

Organs-On-Chip platforms for oncoimmunology (OncoImmuno chips) can be considered as flexible boxes capable of containing the diverse types of immune cells, cancer cells and extracellular matrices, coupled to optimized technologies to monitor the distinct cell behaviors throughout the time (e.g., time lapse microscopy). OncoImmuno chips can be fabricated in a customized manner, depending on the specific type of biological event dealing with or to be investigated. This is a great advantage for using microfluidic devices to study relationships between cancer and immunity.

**FIGURE 10:**
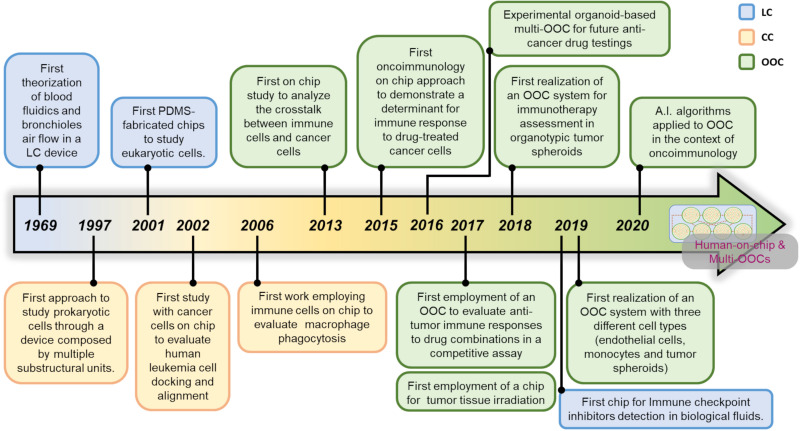
From the dawn of microfluidic devices to the future Human-on-chip for oncoimmunology. Time-line of microfluidic chip evolution from simple Lab-on-Chip (LC) and CC (Cell-on-Chip) devices to Organ-on-Chip (OOC) platforms integrated by AI algorithms for on chip oncoimmunology applications, toward the development of complex multi-organs on chip for in-depth investigations.

The major boost to the development of OOCs, starting from simple LC and CC systems to sophisticated OOC has been the main big goal to design a functional HOC platform. When this will occur, clinical research, cancer research and patient’s personalized medicine and all the research converging to oncoimmunology will certainly benefit of these scientific milestones. The era in which anticancer drug testing to evaluate immune response will be performed in a HOC patient’s personalized avatar (resembling to a human *ad hoc* bio-avatar) can certainly be reached in the long-term future. The application of the deep learning algorithms to the development of these bio-avatars is crucial and can revolutionize the conceptual view and development of such advanced HOC models, particularly in the prediction of anticancer drug effects in immune response to tumors. In conclusion, research to implement advanced OOC platforms, including the OncoImmuno chip is indissolubly linked to the parallel research in neural network systems. The recent encouraging bodies of literature have demonstrated that OncoImmuno chips can be experimentally implemented in an HOC platform with integrated modular microdevices recapitulating a vascular system (Vunjak-Novakovic et al., 2013). Figure 10 delineates the major breakthroughs that led to advanced OOC systems and that can boost the realization of complex HOC integrated by AI algorithms and advanced smart OncoImmuno chips. In this regard, a HOC recapitulating the systemic metastatic spread has been proposed, which exploits the OOC modular concept. In this hypothetical model, there are multiple OOC modules (Blood-Brain barrier on-chip, Bone chip, Lung cancer chip and Liver chip) which have been previously successfully tested as working submodules (Figure 9). These chips can, in the mid-term future, be connected to generate a working metastasis HOC (Sontheimer-Phelps et al., 2019). This represents an encouraging example on the efforts to recreate an immune-oncology HOC by modular addiction of working OOCs *via* separate submodular experiments.

In oncoimmunology, the affordability and accuracy of drug testing methods constitute urgent needs (Rebelo et al., 2016; Dehne et al., 2017). A module-based HOC may represent an optimal way to obtain advanced OOC networks, including immune system, in a fully modular bionetwork. Once finalized, this intricated bionetwork can certainly represent a powerful tool to develop novel anticancer drugs or therapies. The road developing a multi-organ connection is still extremely tangled and far to reach a real HOC system. However, the advent of new encouraging technologies, such as 3D bioprinting (Mota et al., 2020), mathematical models of networks (Cao et al., 2018; Wang Y. et al., 2019) and systems biology (Li et al., 2020), will represent relevant resources to reach this revolutionary scope.

An important issue to deal with is linked by the use of Matrigel for the assembly of microfluidic devices. This gel matrix was initially used in large scale for the recreation of the TME on chip. However, it holds several limitations, such as variability during manufacturing and complexity in composition. In fact, Matrigel is very difficult to handle, often subjected to batch-to-batch concentration variation and there are also some ethical issues related to its use (Aisenbrey and Murphy, 2020). For this reason, the assembly of microfluidic chips is gradually moving toward the use of other biocompatible gels, such as A PEG–maleimide hydrogel scaffold or a fibronectin-derived three-amino-acid peptide Arg-Gly-Asp, which binds to both αvβ3 and αvβ5 integrins, more affordable and ease to assembly. The constant research of new gel matrices suitable for the recapitulation of the TME on chip is of paramount relevance for boosting OOCs and HOCs platform optimization for unsolved oncoimmunology questions.

A final consideration should be outlined on the relationship between the evolution of on chip models and clinical research. If one is oriented to the development of an OncoImmuno chip platform aimed at solving a very specific biological problem, such as the response of immune cells to a tumor, this can require the design of a very simple chip. This will not be a time-consuming process and will not be expensive. On the contrary, if the main purpose is to solve advanced scientific problems, including sophisticated organ simulations, the development of a chip that will satisfy this condition will be time-consuming and very expensive. The developed OncoImmuno chip platform will be useful for basic research purposes rather than to solve a specific clinical problem. The Supplementary Table 3 defines all the major pros and cons to be considered for the use of microfluidic devices in oncoimmunology applications.

## Author Contributions

FM and GS planned, supervised and wrote the manuscript. FM and SA realized the illustrations. SA, AM, AD, LB, and EM reviewed the current literature and contributed to the writing of the manuscript. All authors contributed to the article and approved the submitted version.

## Conflict of Interest

The authors declare that the research was conducted in the absence of any commercial or financial relationships that could be construed as a potential conflict of interest.
