# Supplementary material for: Oncoimmunology Meets Organs-on-Chip
Source: Front Mol Biosci. 2021 Mar 26;8:627454. doi: 10.3389/fmolb.2021.627454 (PMC8032996; doi:10.3389/fmolb.2021.627454)
Supplement: Supplementary file 1 [file Table_1.pdf]

Supplementary Table 1. Examples of experimental OOC platforms used in oncoimmunology.

References	Fabrication method	Main biomaterial	Cells loaded	Gel matrix	Main chip features	OOC Analysis and Evaluations
**Wong, A.P. *et al.* (2008)**	PDMS chip on glass slide, obtained by soft-lithography on a photoresist mould.	PDMS	Human Macrophage cell lines:<br>- BAC1.2F5<br>- LADMAC	Matrigel	Virtual three-chamber chip with no delimiting boundaries, being generated by the gel flow forces during the cell loading phase. Cells are resuspended in Matrigel and partitioned in the side chambers by laminar flow forces.	○ Culture of macrophages in the chambers;<br>○ Qualitative evaluation of the on chip migration between the two macrophage cell lines;<br>○ Quantitation of live cells by Propidium Iodide labelling.
**Sung J.H. and Shuler M.L. (2009)**	PDMS chip obtained by soft-lithography processes coupled to stereo lithography. Glass is used as a cover after the cell loading process.	PDMS	- HCT-116 (colon cancer cell line);<br>- HepG2 (hepatoma cell line to mimic liver cells);<br>- Kasumi-1 (Myeloblasts to mimic bone marrow cells)	Matrigel	Three chambers for HCT-116 cells, HepG2 cells and Kasumi-1 cells. The chambers are interconnected by single fluidic channels mimicking the fluid flow between the three systems. The medium is perfused by a pump at a fixed flow rate. The chip is also connected to a bubble trap system.	○ Drug toxicity studies;<br>○ tumour cell and liver cell morphology;<br>○ Tumour cell viability and growth.
**Businaro L. *et al.* (2013)**	PDMS chip on glass slide, obtained by soft-lithography processes on a photoresist mould obtained by stereo lithography.	PDMS	- Murine cancer cells: B16.F10 (metastatic melanoma);<br>- Murine splenocytes cells derived from IC or NIC.	None used	A center channel (1000 µm large) adjacent to two side cell compartments (1000 µm large) used to load splenocytes and melanoma cells. These two chambers and center channels were connected by two microchannel arrays allowing the exclusive flow-through of splenocytes.	○ Counts of splenocytes and immune cells in IC vs NIC conditions;<br>○ Interactions between splenocytes and cancer cells in IC vs NIC conditions by time-lapse microscopy;<br>○ Real-time monitoring of B16 cells extravasation in microchannels in IC vs NIC conditions by time-lapse microscopy;<br>○ Quantitation of single splenocyte motility in IC vs NIC conditions;<br>○ Extrapolation of tracking profiles of splenocytes in IC vs NIC conditions;<br>○ Quantitation of cell-cell interaction times in IC vs NIC conditions.
**Pavesi A. *et al.* (2017)**	PDMS chip on glass slide, obtained by soft-lithography and stereo lithography.	PDMS	- Human tumour cells: HepG2-Env tumour cells;<br>- Human immune cells: engineered T cells expressing cancer-specific T cell receptors.	Collagen	Three chambers separated by adjacent trapezoidal boundaries which avoid gel to extravasate into the flanking chamber. T cells and cancer cells are loaded into the two chambers. The third chamber is filled with medium only and serves as a flow stabilizer system.	○ Recapitulation of the tumour hypoxia and inflammation on chip;<br>○ Evaluation and quantitation of T cell cytotoxicity by time-lapse microscopy.

**Fang G. *et al.* (2019)**	3D bioprinting technology.	Ink-Grey Resin	Human studies:<br>- Tumour spheroids generated by MCF-7 breast cancer cell line;<br>- Primary cells: normal human dermal fibroblasts.<br><br>Mouse studies:<br>- Tumour spheroids derived from 4T1.2 breast cancer cells (Balb/C background);<br>Primary cells: fibroblasts from Balb/C mice.	Agarose	Circular microdevice with multiple microwells (about 500 µm) used for the development and growth of tumour spheroids. A peculiar feature of this microdevice is the formation of a liquid dome that establish a differential growth of the spheroid in function of their position inside a specific microwell.	○ Real-time monitoring of the tumour cells plus fibroblasts organoids growth in the microwells in Control vs Latrunculin-treated chips;<br>○ Quantitation of the organoid growth & viability as well as fibroblast infiltration in Control vs Latrunculin-treated microdevices
**Beckwith A.L. *et al.* (2019)**	3D bioprinting technology.	Methacrylate-based Resin	Fresh biopsy from non-small cell lung cancer patients.	None used	Microdevice (25mm x 38mm) equipped with a chamber for the insertion of the biopsy sample. It is also featured with inlet and outlet ports for medium and with a bubble trapping system, all managed by a pump system. The biopsy chamber is connected to an inlet and two outlet microchannels which constantly perfuse the tumour tissue with medium.	○ Real-time monitoring of TILs PD-1 expression in tumour biopsy by fluorescence microscopy;<br>○ Real-time monitoring of tissue viability by fluorescence microscopy.

Legend:

PDMS, Poly-(dimethyl)-siloxane; IC, Immunocompetent splenocytes; NIC, Non-immunocompetent (IRF-8 knock-out) splenocytes; 3D, three dimensional; TIL, Tumour-infiltrating Lymphocyte.
