# Supplementary material for: Oncoimmunology Meets Organs-on-Chip
Source: Front Mol Biosci. 2021 Mar 26;8:627454. doi: 10.3389/fmolb.2021.627454 (PMC8032996; doi:10.3389/fmolb.2021.627454)
Supplement: Supplementary file 2 [file Table_2.pdf]

Supplementary Table 2. Examples of commonly used software platforms for cell migration tracking and image-assisted post-analysis.

Commercial platforms[$]			Open source platforms[#]		
**Software Name**	**Source**	**Notes**	**Software Name**	**Source**	**Notes**
**Imaris Track**	https://imaris.oxinst.com/	Free trial available. Specialized for particle tracking inside cells.	**Cell Profiler**	https://cellprofiler.org/	Used for migration cell tracking, but particularly specialized in particle tracking inside cells.
**Image Pro Plus**	https://www.mediacy.com/	High performance cell tracking, requiring very performant systems. Free trial available.	**Vaa 3D**	https://alleninstitute.org/	Limited to neuronal cell tracking and post-image analysis of neuronal cells.
**Velocity**	https://www.velocitysoftware.com/	Can be directly connected to microscope systems for real-time cell migration tracking.	**Bioimage XD**	https://www.bioimagexd.net/	Particularly used for advanced image processing. High versatility for cell tracking options. Multi-purpose post-processing tool for bioimaging.
**Amira**	https://www.thermofisher.com/	Free trial available. Large range of cell tracking options.	**Icy**	http://icy.bioimageanalysis.org/	Particularly used for particle tracking inside the cells. Requires high performance systems to work properly. Discrete number of plugins available.
**MetaMorph**	https://www.moleculardevices.com/	Particularly specialized in single image frame post-analysis.	**Fiji**	https://fiji.sc/	Very similar to ImageJ: the source code is indeed derived from this software.
**HoloMonitor App Suite**	https://phiab.com/	Real-time and label-free single cell tracking software to be done with Holomonitor microscope. Exploits holographic features of the cells.	**ImageJ**	https://imagej.nih.gov/ij/	Cell tracking analysis available as plug-in in automated or manual settings (TrackMate). Large number of plug-ins available and made by users.

**Tracking Tool Pro**	https://gradientech.se/	Similar to the ImageJ TrackMate plug-in, but with a user-friendly GUI. Automatically tracks both labeled or unlabeled cells. Manual cell tracking option available for free.	**Cell Hunter**[&]	Comes, M.C. *et al.*, 2020b	MatLab code available on request. Specifically adapted to recognise immune and cancer cells. Specific machine learning code for the automated computation of the cell-cell interaction times.
			**Knime**	https://www.knime.com/	Some packages are not freely available. On-demand generation of specific software upon payment.
			**Farsight**	http://biii.eu/	High number of available cell tracking options.
			**Chemotaxis and Migration Tool**	https://www.ibidi.com/	Software freely available. Cell tracking analysis specific for proprietary microfluidic devices.
			**CellTracker**	http://www.celltracker.website/	MatLab-based automated cell tracking algorithms. Similar to the Cell Hunter GUI interface. Particularly useful for immuno-oncology studies.

Legend

$ Ease of use, with user-friendly GUIs. A free time-limited trial often available to be used. A permanent free version, with limited options, is available for some programs.

Completely transparent and freely availabe algorithms or programs, but often suitable for expert users.

& Extended reference data: Comes MC, Filippi J, Mencattini A, Corsi F, Casti P, De Ninno A, Di Giuseppe D, D'Orazio M, Ghibelli L, Mattei F, Schiavoni G, Businaro L, Di Natale C, Martinelli E. Accelerating the experimental responses on cell behaviors: a long-term prediction of cell trajectories using Social Generative Adversarial Network. Sci Rep. 2020 Sep 24;10(1):15635. doi: 10.1038/s41598-020-72605-3. PMID: 32973301; PMCID: PMC7519062.
