# Supplementary material for: Oncoimmunology Meets Organs-on-Chip
Source: Front Mol Biosci. 2021 Mar 26;8:627454. doi: 10.3389/fmolb.2021.627454 (PMC8032996; doi:10.3389/fmolb.2021.627454)
Supplement: Supplementary file 3 [file Table_3.pdf]

Supplementary Table 3. Considerations to take into account for the use of microfluidic chips in oncoimmunology.

Advantages	Disadvantages
Versatility of use for oncoimmunolgy applications:<br>○ drug validations;<br>○ immune checkpoint inhibitors;<br>○ interactions between specific components of the immune system and cancer (i.e., CAFs and immune cell subsets);<br>○ Focused studies on the disparate aspects of the TME mimicry.	Structurally complex devices can affect the experimental reproducibility and compromise the TME mimicry. On the contrary, simple devices are not suitable to recapitulate with high fidelity the TME scenario.
Soft lithography-based fabrication techniques for PDMS chips allow an *ad hoc* chip compartmentalization and the storage of a Master for each type of chip.	The overall chip fabrication processes (chip design, and experimental validation) can significantly increase with the structural complexity of the chip.
Minimization and optimization of the use of mice employed for *in vivo* oncoimmunology-based experiments (i.e., non-immunocompetent animals) and the number of cells to be loaded on chips to compliant with the 3R rules.	Need for parallel validation with *in vivo* or *ex vivo* systems, especially for new prototypes.
Instrumental versatility: each chip can be coupled to disparate simple, smart or advanced microscopy and holographic systems.	Specific instrumental control requirements required (strict control of temperatures, CO_2/O_2 levels, humidity) to avoid liquid evaporation issues due to microscale volumes used to load cells.
Execution of highly automated and fully programmable experiments: growing availability of specific mathematical algorithms for the monitoring of immune cells or cancer cells in microfluidic platforms.	The implementation of specifically complex problem-solving algorithms may require the use of dedicated and specialized staff, often difficult to recruit.
Computation of new analytical typologies of data arrays (cell tracking profiles, cell-cell interaction parameters and kinematic descriptors strictly associated to the behaviour of immune cells and cancer cells inside an OncoImmuno chip).	Reference standards for clinical validations of immunological determinants still not available for microfluidic devices. Urgent need to structure an immuno-oncological body devoted to the definition of standardization of chips to be used in oncoimmunology investigations.

The use of microscopy systems to perform parallel and distinct time-lapse acquisitions on multiple chambers and sub-compartments structures of the chip (i.e., different regions of the TME on chip can be followed as internal replicates).	Fabrication of microdevices often too expensive in terms of processes and machinery maintenance.
Cancer cells can be monitored on chip after during the time-lapse via targeted FBF detection of the cell area to evaluate their killing extent under the presence of an apoptotic drug or the adhesion to immune cells.	Research of valid alternative to the use of Matrigel to recapitulate the TME on microfluidic devices. Matrigel is often expensive and subjected to batch-to-batch variations.
Creation of simplified OncoImmuno chip to study single components of the TME in an easier way, without losing their functional *in vivo* properties.	Some immune cell subsets (i.e., eosinophils, basophils, dendritic cells) are particularly subjected to mechanical stress during their loading which can compromise the cell's vitality during the time-lapse acquisition.
Generation of an advanced system that recapitulates the relationships between immune system and cancer with high fidelity.	Microscope systems must be equipped with efficient, automated and in-depth focal systems.

Legend

CAF, Cancer Associated Fibroblast; FBF, Frame-by-Frame; TME, Tumour Microenvironment.
